# DNA Methylation in Blood Samples from Malignant Mammary Tumors of Companion Dogs: A Pilot Study

**DOI:** 10.34133/csbj.0104

**Published:** 2026-05-14

**Authors:** Keunhwan Jang, Geunwoo Jeon, Jungwoo Han, Seung-Bum Cho, Suyeon Kim, Songju Oh, Ha-Jung Kim

**Affiliations:** ^1^ Department of Internal Medicine, College of Veterinary Medicine, Chonnam National University, Gwangju 61186, South Korea.; ^2^ BK 21 project team, College of Veterinary Medicine, Chonnam National University, Gwangju 61186, South Korea.

## Abstract

Canine mammary gland tumors (cMGTs) are neoplasms arising from mammary epithelial or supporting tissues and account for approximately 50% to 70% of all tumors in intact female dogs. Epigenetic regulation, such as DNA methylation, has been investigated to elucidate the molecular mechanisms of cMGTs. This study explored whether DNA methylation alterations in peripheral blood reflect tumor-related epigenetic patterns. Eighteen client-owned dogs were enrolled, including 10 with cMGTs and 8 healthy controls. Although no individual genes reached statistical significance after multiple testing correction, exploratory gene ontology analysis identified 22 biological processes showing potential enrichment, including 2 hypermethylated and 20 hypomethylated regions. Comparative analysis between canine and human datasets revealed 91 overlapping hypomethylated genes displaying consistent patterns across both species. The top enriched gene ontology terms were morphogenesis of an epithelium and cell growth. These findings provide strictly exploratory and hypothesis-generating evidence that blood-based DNA methylation profiling may capture tumor-associated systemic epigenetic alterations in cMGTs.

## Introduction

A tumor represents uncontrolled cell growth and proliferation, which is driven by variations in the expression of specific genes including oncogenes, tumor suppressor genes, and DNA repair genes [1]. Variations in gene expression driving tumorigenesis involve either direct DNA sequence alterations (e.g., point mutations, deletions, and translocations) or epigenetic modifications, such as DNA methylation, which regulate gene expression without changing the underlying sequence [2,3].

DNA methylation involves the transfer of a methyl group to the fifth carbon of a cytosine residue by DNA methyltransferases [3]. This modification predominantly occurs in CpG islands, which harbor approximately 70% of gene promoters [3,4]. Methylation represses transcription directly by blocking transcription factor binding and indirectly by recruiting methyl-CpG-binding proteins and chromatin remodeling complexes [5,6]. Aberrant epigenetic regulation, particularly DNA methylation, has emerged as a key mechanism in neoplastic development [7–10]. In human breast cancer, environmentally mediated hypermethylation of tumor suppressor genes serves as an early biomarker of carcinogenesis. Consequently, DNA methylation profiling offers promising clinical strategies for early diagnosis, targeted treatment, and prognostic evaluation [7].

In 2024, approximately 970,000 new cancer cases were diagnosed in women in the United States, with about 32% (310,000 cases) being breast cancer [8]. The incidence rate of female breast cancer has been gradually rising at an annual rate of approximately 0.6% since the mid-2000s [9]. The mortality rate of female breast cancer peaked in 1989 and declined by 42% by 2021, driven by early detection [8]. For early detection, a multigene panel using methylated cell-free DNA extracted from blood demonstrated a 95% sensitivity in detecting breast cancer, emphasizing the potential for noninvasive breast cancer diagnosis [10]. Gene therapy, which targets the regulation of DNA expression in tumor cells, involves introducing genetic material into target cells via a vector, followed by gene correction, insertion, or inhibition, as demonstrated in humans [11]. In addition, veterinary medicine continues its research on understanding canine mammary gland tumors (cMGTs) through DNA methylation [12–14]. cMGTs are neoplasms of mammary glandular tissues or supporting structures, comprising over 50% to 70% of tumors in intact female dogs [15]. Approximately 50% of these tumors in canines are malignant, with an increased risk in nonspayed female dogs over 7 years of age [15,16]. It is capable of metastasizing to surrounding lymph nodes, lungs, kidneys, and liver [17]. These tumors are staged based on tumor size, involvement of regional lymph nodes, and distant metastasis, in accordance with the tumor-node-metastasis (TNM) staging system, and histological grades are determined by tubule formation, nuclear pleomorphism, and mitotic count per high-power field [18]. Prognosis varies depending on tumor stage and grade [18]. While complete surgical excision substantially extends the median survival time (872 d) compared to incomplete excision (70 d), advanced diagnostic and therapeutic markers are still required [19]. Recently, DNA methylation profiling of peripheral blood mononuclear cells (PBMCs) in dogs with cMGTs identified hypermethylated candidate genes, suggesting that circulating immune cells may provide valuable epigenetic prognostic markers [14].

The present study aims to characterize genome-wide DNA methylation patterns in the peripheral blood of patients with cMGT compared to healthy controls. By identifying differentially methylated regions (DMRs) associated with cMGTs and comparing these epigenetic signatures to human breast cancer datasets, we seek to elucidate shared tumorigenic mechanisms across species. Ultimately, these findings may contribute to the development of noninvasive diagnostic, prognostic, and therapeutic strategies for cMGTs.

## Results

### Number of hypermethylated and hypomethylated CpGs in the cMGT group compared with the control group

**Fig. 1.**
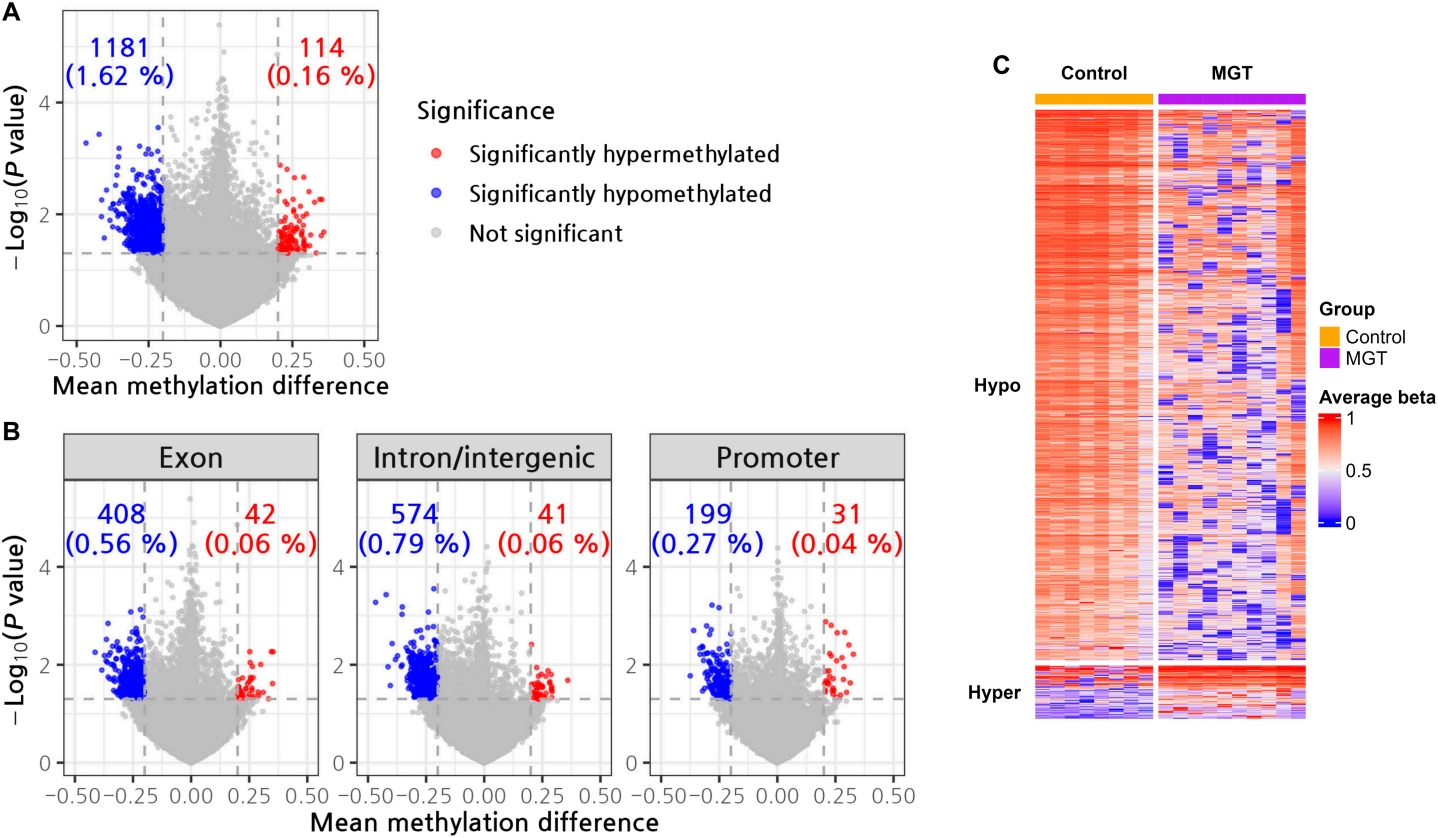
(A) Differentially methylated region (DMR) analysis. A total of 1,295 methylated CpGs were found in the canine mammary gland tumors (cMGT) group compared to the control groups, comprising 114 hypermethylated and 1,181 hypomethylated genes. (B) the 1,295 methylated CpGs revealed 450 CpGs in exons (hyper, 42; hypo, 408), 615 CpGs in introns/intergenic regions (hyper, 41; hypo, 574), and 230 CpGs in promoters (hyper, 31; hypo, 199), indicating methylated CpGs in each region. The delta mean and *P* value were obtained from the comparison of the average for each group plotted as the volcano plot (*x* axis, delta mean; *y* axis, −log_10_ *P* value). (C) A heatmap illustrates the divergence between the cMGTs and the control groups.

Based on the differential methylation analysis of the enrolled dogs (Table 1), 1,295 differentially methylated CpG sites were identified in the cMGT group compared to the control group, comprising 114 hypermethylated and 1,181 hypomethylated CpGs. A volcano plot illustrates the distinct divergence in methylation patterns between the 2 groups (Fig. 1A). When classified by genomic region, these 1,295 CpGs were distributed across exons (450 CpGs; 42 hyper and 408 hypo), introns and intergenic regions (615 CpGs; 41 hyper and 574 hypo), and promoters (230 CpGs; 31 hyper and 199 hypo). To visually represent the distinct methylation patterns, we created 3 volcano plot charts, illustrating the divergence between the cMGTs and the control groups (Fig. 1B).

**Table 1.** Characteristics of all participants

Signalment		Total (*n* = 18)	
		Canine mammary gland tumors (*n* = 10)	Control (*n* = 8)
Sex, *n* (%)			
	Intact female	6 (60)	5 (62.5)
	Spayed female	4 (40)	3 (37.5)
Age, *n* (%)			
	<4 y	1 (10)	4 (50)
	4–7 y	0 (0)	0 (0)
	>7 y	9 (90)	4 (50)
Breed, *n* (%)			
	Maltese	3 (30)	1 (12.5)
	Pomeranian	0 (0)	1 (12.5)
	Beagle	1 (10)	4 (50)
	Shih-tzu	1 (10)	1 (12.5)
	Jindo	1 (10)	0 (0)
	Border Collie	1 (10)	0 (0)
	Yorkshire Terrier	2 (20)	0 (0)
	Mixed	1 (10)	1 (12.5)

A heatmap illustrates the divergence between the cMGTs and the control groups (Fig. 1C).

### Gene ontology overrepresentation analysis in the cMGT group

**Fig. 2.**
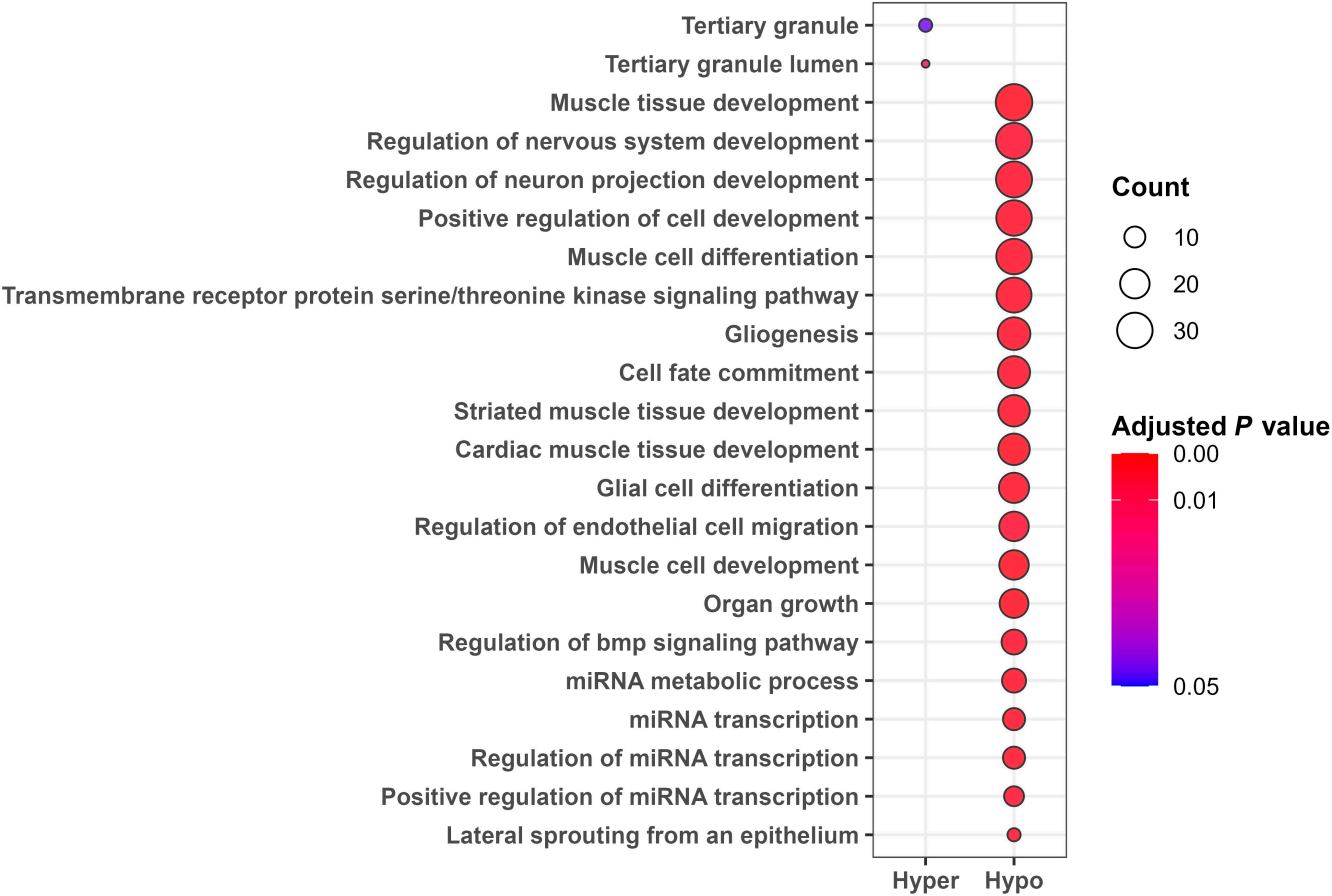
Through gene ontology overrepresentation analysis, significant gene ontology information surrounding methylated regions in the canine mammary gland tumors (cMGT) group has been summarized. A total of 22 significant gene ontology information, with 2 in the hypermethylation region and 20 in the hypomethylation region.

Gene ontology (GO) overrepresentation analysis was conducted to summarize the biological functions associated with the DMRs. A total of 22 GO terms showed significant enrichment, including 2 terms associated with hypermethylated regions and 20 terms associated with hypomethylated regions (Fig. 2). These enriched terms were categorized into primary biological groups based on functional similarities (Tables 2 and 3).

**Table 2.** Gene ontology overrepresentation analysis in the hypermethylated gene

	Gene ontology	*P* value
Neutrophil component	Tertiary granule lumen	0.012*
	Tertiary granule	0.046*

Values with statistical significance are indicated in bold. *P < 0.05.

**Table 3.** Gene ontology overrepresentation analysis in the hypomethylated gene

	Gene ontology	*P* value
Muscle development	Cardiac muscle tissue development	0.001**
	Muscle cell development	0.001**
	Muscle tissue development	0.001**
	Striated muscle tissue development	0.001**
	Muscle cell differentiation	0.002**
Cell proliferation and migration	Organ growth	0.001**
	Cell fate commitment	0.001**
	Lateral sprouting from an epithelium	0.002**
	Positive regulation of cell development	0.002**
	Regulation of endothelial cell migration	0.003**
miRNA regulation	Regulation of miRNA transcription	0.002**
	miRNA transcription	0.002**
	miRNA metabolic process	0.002**
	Positive regulation of miRNA transcription	0.003**
Signaling pathway	Transmembrane receptor protein serine/threonine kinase signaling pathway	0.002**
	Regulation of BMP signaling pathway	0.002**
Nervous system	Glial cell differentiation	0.002**
	Gliogenesis	0.002**
	Regulation of neuron projection development	0.002**
	Regulation of nervous system development	0.003**

Values with statistical significance are indicated in bold. *P < 0.05 and **P < 0.01.

miRNA, microRNA; BMP, bone morphogenetic protein.

### GO network analysis in the cMGT group

**Fig. 3.**
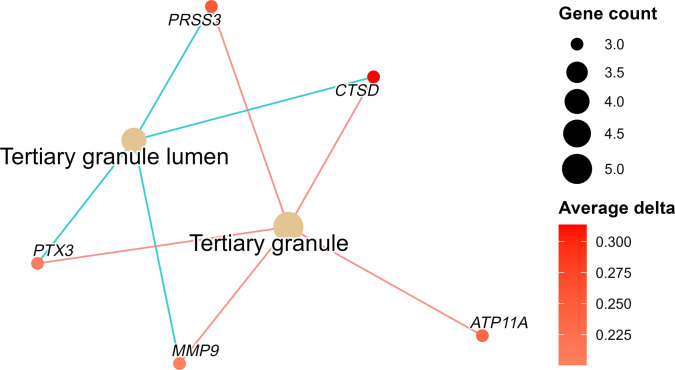
Gene ontology network in the hypermethylated differentially methylated regions (DMRs). Two gene ontology information sets were identified, and networks were generated to display 5 related genes.

**Fig. 4.**
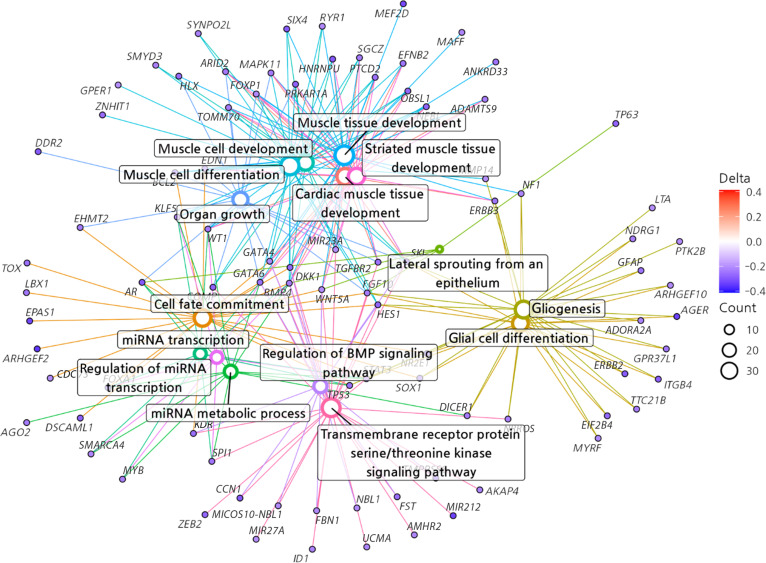
Gene ontology network in the hypomethylated differentially methylated regions (DMRs). A total of 20 gene ontology information sets were identified, and networks were generated to display related genes. BMP, bone morphogenetic protein.

Utilizing the GO data and differentially methylated genes, network analyses were constructed to visualize the interactions and functional relationships among the hypermethylated and hypomethylated genes in the cMGT group (Figs. 3 and 4).

### A comparative analysis of DNA methylation in canine mammary tumors and human breast cancer

**Fig. 5.**
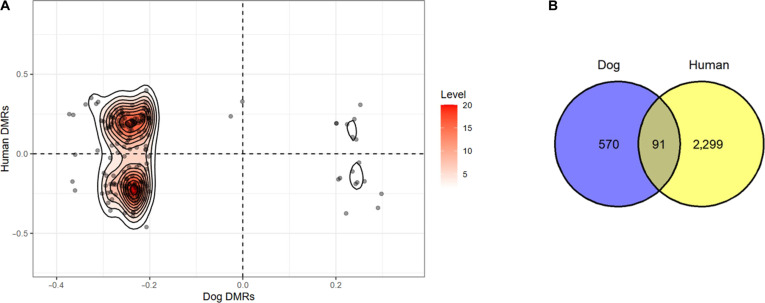
Using differentially methylated regions (DMRs), significant hypomethylated genes were identified in the third quadrant of the interactive plot for both canine mammary tumors and human breast cancer (A), with 91 of them being commonly hypomethylated in both species (B).

To explore cross-species epigenetic similarities, we compared the differentially methylated CpG sites from our canine dataset to a human dataset representing normal breast tissue at risk of malignant transformation. The correlation analysis revealed substantial DNA methylation alterations in both the cMGT and human datasets. Notably, 91 commonly hypomethylated orthologous genes were identified across both species (Fig. 5B).

### GO analysis of hypomethylated genes in canine, human, and commonly identified genes

**Fig. 6.**
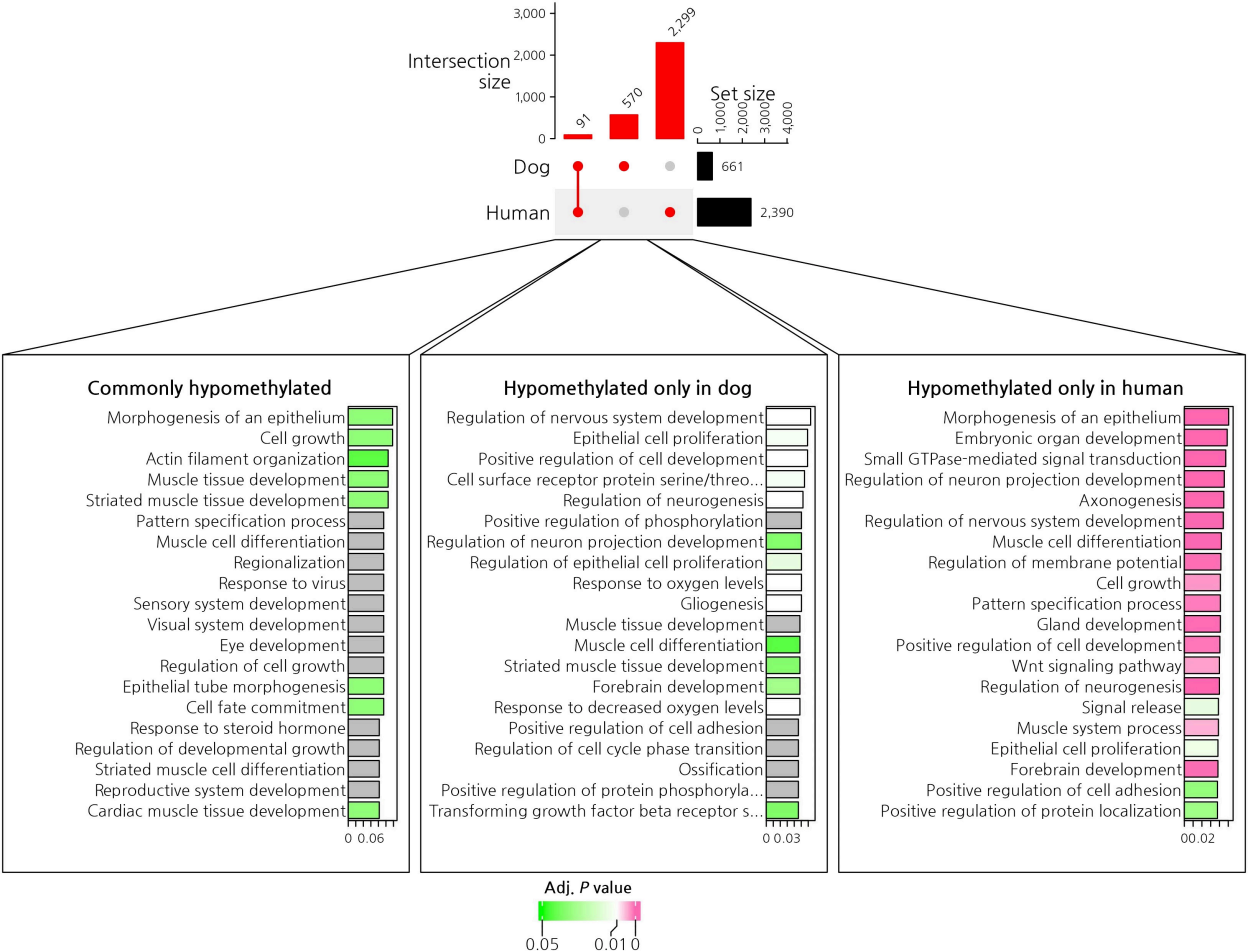
Gene ontology (GO) analysis of the 91 commonly hypomethylated genes revealed significant enrichment, with morphogenesis of an epithelium and cell growth identified among the top 20 gene ontology information sets. GTPase, guanosine triphosphatase.

GO analysis was performed to identify the top 20 enriched biological processes for hypomethylated genes in dogs, humans, and the shared gene set. Among the 91 commonly hypomethylated genes, significant enrichment was observed for pathways related to the morphogenesis of an epithelium and cell growth (Fig. 6).

## Discussion

**Fig. 7.**
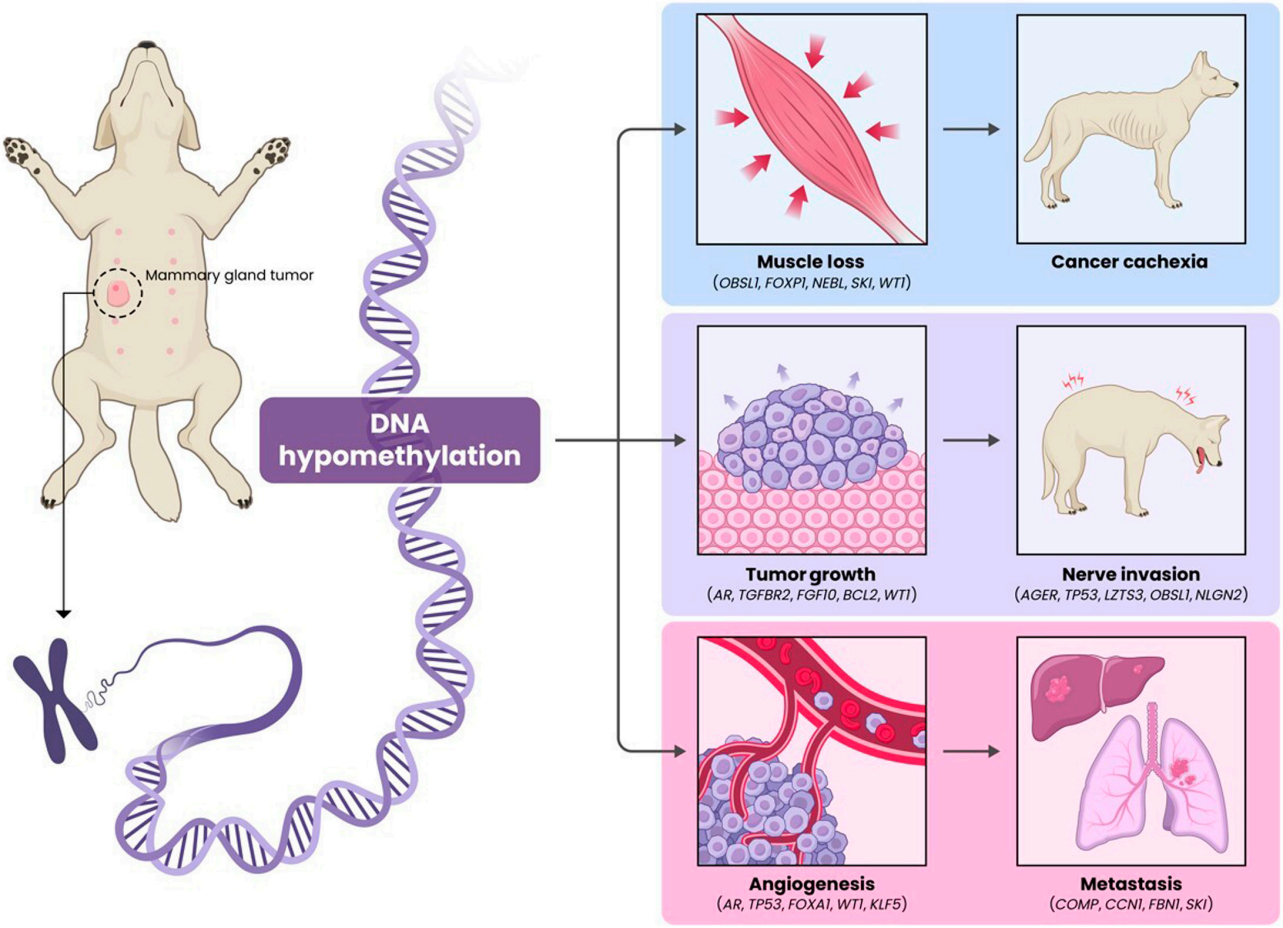
Proposed model of canine mammary gland tumors (cMGT) progression based on exploratory DNA methylation patterns. Hypomethylated candidate genes identified in the cMGT group (compared to controls) may contribute to pathways associated with muscle loss, tumor growth, nerve invasion, angiogenesis, and metastasis, potentially leading to cachexia, pain, and metastatic organ dysfunction. These candidate epigenetic alterations represent exploratory indicators that warrant further validation for diagnostic and prognostic applications in cMGTs.

In the present study, we discovered important information about 6 GO categories. Among them, 1 is hypermethylated regions of cellular components, and 5 are hypomethylated regions of biological processes. In human medicine, several studies have investigated the prognostic implications of this cellular component or biological process in tumors. This study has revealed, from an epigenetic perspective through methylation analysis, the expression of these genes, thereby offering a new perspective on the diagnosis, treatment, and prognosis of cMGTs (Fig. 7.).

In the present study, DNA methylation was conducted using a less invasive approach, which involved obtaining peripheral blood, rather than invasive tissue sampling. Following this trend, recent studies have utilized DNA methylation analysis of PBMCs for early diagnosis of breast cancer in humans [20]. Similarly, DNA methylation analysis of PBMC in the diagnosis of cMGTs revealed 4 hypermethylated genes (*BACH2*, *SH2D1A*, *TXK*, and *UHRF1*) associated with T, B, and natural killer cell activity, enabling differentiation between normal and tumor, as well as benign and malignant tumors [14]. Furthermore, through saliva samples for DNA methylation analysis, the deleted in colorectal cancer netrin 1 receptor (*DCC*) gene has been reported as a method for early detection of superficial hypopharyngeal cancer in high-risk patients [21].

In the present study, tertiary-granule-associated genes were hypermethylated, leading to down-regulation of gene expression. While previous literature has reported increased expression of these genes within the local tumor microenvironment to facilitate metastasis, the hypermethylation observed in our peripheral blood samples may paradoxically reflect a systemic immune exhaustion or suppressive phenotype in the host [22]. Tertiary granules are a constituent component of neutrophils containing matrix metalloprotease 9, facilitating rolling interaction with the activated endothelium [23]. In several studies, neutrophils have been reported to have a dual impact on the cancer microenvironment in human [24]. In terms of both tumor growth inhibition and metastasis suppression, neutrophils exert their effects through the expression of antibody-dependent cellular cytotoxicity, which kills cancer cells, and the production of H_2_O_2_, thereby inhibiting tumor growth and metastasis [25]. In the present study, several tertiary-granule-associated genes, including *CTSD*, *PTX3*, *ATP11A*, *PRSS3*, and *MMP9*, were identified as hypomethylated candidates within enriched GO terms, although no individual genes reached significance after multiple testing correction, and previous studies have reported that increased expression of these genes is associated with enhanced tumor growth and metastasis in various cancers, suggesting that tertiary-granule-related pathways may also be involved in the progression of cMGTs [26–30]. Despite the present study result contradicting the published research, there are studies linking DNA hypermethylation to increased gene expression [31,32]. Therefore, further research is needed.

Cancer-associated cachexia accompanies high rates of mortality, involving muscle loss, body weight loss, and inflammation [33,34]. In several studies, decrease in muscle-anabolism-related gene expression has been reported in cancer-associated cachexia [35,36]. In the present study, muscle-development-related genes (*OBSL1*, *FOXP1*, *NEBL*, *SKI*, and *WT1*) were identified as hypomethylated candidates within enriched GO terms, and previous studies have reported increased expression of these genes in association with tumor progression [37–41]. Specifically, forkhead box P1 (FOXP1) has been reported as a transcriptional repressor of skeletal muscle gene expression, inducing muscle wasting and worsening the prognosis of tumor in mice [40]. Therefore, it is strongly suggested that cancer-associated cachexia could be implicated through *FOXP1* in cMGTs.

Uncontrolled cell proliferation and migration have a substantial impact on tumor growth and metastasis, making them crucial for assessing tumor prognosis [42]. In the present study, the genes *AR*, *TGFBR2*, *FGF10*, *BCL2*, and *WT1* were identified as hypomethylated candidates, and previous studies have reported increased expression of these genes in association with tumor progression [41,43–46]. In particular, the androgen receptor (*AR*) gene, which exhibits the most hypomethylation, is expressed in 60.5% of human breast cancers [46]. As a type I nuclear receptor, AR binds to androgens that have passed through the cell membrane, thereby influencing the DNA transcription of target genes [47]. It promotes or inhibits cellular differentiation, proliferation, apoptosis, or angiogenesis depending on the bioavailability of estrogens [48]. In human retrospective studies, high expression of AR and AR/estrogen receptor ratio was associated with a recurrence rate of 91.7% in ductal carcinoma in situ of the breast, indicating the potential of AR as a prognostic indicator [49]. In human medicine, while immunohistochemical methods for assessing AR expression have been widely adopted, there is a recent trend of attempting less invasive liquid biopsy methods using plasma to measure AR levels [50]. Therefore, based on these results, it is evident that less invasive methods, such as liquid biopsy using blood samples, can be applied to evaluate prognostic factors in cMGTs.

MicroRNAs (miRNAs) are small RNA molecules that are noncoding and single stranded, and they control gene expression posttranscriptionally by interacting with mRNA [51]. miRNAs regulate cell division, proliferation, differentiation, apoptosis, and angiogenesis [52]. The differential expression of miRNAs has been observed in numerous cancers, where they can act as either oncogenes or tumor suppressors under certain conditions [53]. In the present study, *AR*, *TP53*, *FOXA1*, *WT1*, and *KLF5* were identified as hypomethylated candidate genes, and previous studies in human medicine have reported overexpression of these genes to be associated with tumor progression [41,46,54–57]. In particular, tumor protein p53 (TP53) acts as a tumor suppressor, regulating cell division by preventing cells from proliferating excessively. TP53 repairs damaged DNA or induces apoptosis in cells with irreparable DNA damage, thereby preventing the development of tumors [57]. In human breast cancer, *TP53* mutations account for 30% to 35% of cases, and research has been conducted on survival prediction in human breast cancer through *TP53* mutation status [58–61]. However, it has also been reported that TP53 regulates the processing of precursor miRNAs, and within this miRNA network of TP53, certain miRNAs can down-regulate both the levels and activity of the TP53 protein by directly suppressing its expression in human tumors [62,63]. In the present study, nominal hypomethylation of the *TP53* gene has been discovered. Although TP53 acts as a well-established tumor suppressor when fully functional, it also regulates the processing of precursor miRNAs. Aberrant hypomethylation of *TP53* in circulating cells may alter this complex miRNA network, potentially leading to an indirect down-regulation of TP53 protein activity or reflecting genomic instability. However, this remains a highly speculative mechanism requiring further targeted investigation.

Enhanced signaling pathways promote tumor growth and invasion [64,65]. Receptor serine/threonine kinase signaling increases vascular endothelial growth factor C expression, inducing angiogenesis and metastasis, and bone morphogenetic protein signaling, interacting with transforming growth factor-β (TGF-β), has been reported to promote tumor growth and invasion [64,65]. In the present study, *COMP*, *CCN1*, *FBN1*, and *SKI* were identified as hypomethylated candidate genes, and previous studies in humans have reported these genes to be associated with tumor progression, whereas the role of follistatin (FST) in cMGTs remains to be clarified [39,56,66–68]. FST is a secreted extracellular regulatory protein that acts as an antagonist to the TGF-β superfamily [69]. Decreased expression of FST has been reported to result in increased proliferation, angiogenesis, and metastasis, leading to poor prognosis in human breast cancer [68,70]. Because it has not been previously reported that *FST* gene regulation and its relationship with cMGTs differ from those of the present study in human breast cancer, it indicates the need for further research.

In the present study, the nervous-system-related genes *AGER*, *TP53*, *LZTS3*, *OBSL1*, and *NLGN2* were identified as hypomethylated candidate genes, and previous studies in human cancers have reported overexpression of these genes to be associated with tumor progression [37,57,71–74]. Among the associated genes, neuroligin 2 (NLGN2), a family of cell adhesion molecules, is expressed in glial cells in the central nervous system, playing a role in the formation, maturation, and function of synaptic structures [74,75]. Glial cells, including microglia, astrocytes, and oligodendrocytes, help support, connect, and protect the neurons of the central and peripheral nervous systems [76]. In recent studies, reports indicate that the activation and accumulation of glial cells play an important role in metastatic breast cancer, as well as in perineural metastasis in pancreatic cancer [77,78]. In human breast cancer, elevated NLGN2 expression is significantly correlated with larger tumor size and lymph node metastasis [73]. Although there is currently no report on the relationship between cMGTs and the *NLGN2* gene, based on the present study results, investigating *NLGN2* expression could be utilized as a prognostic indicator for cMGTs.

In the present study, DNA methylation analysis of both human breast cancer and cMGTs revealed 91 commonly hypomethylated genes, among which *FOXP1*, *NEBL*, *WT1*, *TGFBR2*, and *BCL2* were of particular interest. These genes are known to play important roles in tumor progression, including cell proliferation, differentiation, and survival [38,40,41,43,45]. *FOXP1* has been implicated in cancer progression by acting as a transcriptional repressor in muscle-related genes, while *NEBL* has been associated with cytoskeletal organization, potentially influencing tumor cell motility [38,40]. *WT1* is known for its dual role as both a tumor suppressor and an oncogene, depending on cellular context, and its aberrant expression has been linked to breast cancer progression [41]. In addition, *TGFBR2*, a key regulator of the TGF-β signaling pathway, has been associated with tumor invasion and metastasis, while *BCL2* is widely recognized for its role in inhibiting apoptosis, promoting tumor cell survival [43,45]. The consistent hypomethylation of these genes in both human and canine tumors suggests potential shared epigenetic mechanisms underlying mammary tumor development across species. However, it is important to note that this overlap was identified at the gene level. Due to the cross-species application of the array, we cannot definitively conclude that identical homologous regulatory regions or precise nucleotide sites share the exact same methylation patterns. Further research into their functional roles could enhance our understanding of mammary tumor pathophysiology and identify novel therapeutic targets for both human and veterinary oncology.

When interpreting these results, the biological origin of the analyzed samples must be taken into account. Because genomic DNA was extracted from the buffy coat layer, the observed methylation patterns predominantly reflect the epigenetic state of circulating immune cells, representing the host’s systemic immune response, tumor-induced inflammation, or immune evasion mechanisms. While this differs from direct tumor-specific epigenetic alterations, evaluating systemic circulating cells offers a distinct clinical and analytical advantage. Solid mammary tumors frequently exhibit pronounced intratumor heterogeneity, which can introduce substantial sampling bias and variability depending on the precise biopsy site. In contrast, peripheral blood provides a highly homogeneous and easily accessible sample that captures a comprehensive systemic footprint of the tumor’s impact [79–81]. Whether these systemic epigenetic shifts are primary drivers of tumorigenesis or secondary consequences of tumor–host interactions, their consistent detection highlights their strong potential as robust, noninvasive prognostic indicators for cMGTs, circumventing the limitations of traditional tissue biopsies.

Beyond the systemic origin of these epigenetic signatures, their specific genomic distribution provides further insight into their regulatory mechanisms. While differential methylation in promoter regions is classically associated with direct transcriptional silencing, our results showed notable alterations primarily in introns and intergenic regions. Recent epigenetic evidence suggests that these nonpromoter regions often contain crucial regulatory elements, such as enhancers and noncoding RNAs [82,83]. Thus, epigenetic dysregulation in these areas can substantially disrupt long-range gene networks driving neoplastic transformation [84].

In addition, several studies have investigated anticancer drugs targeting DNA methylation [85,86]. The DNA hydroxyl methylation inhibitor, AGI-5198, can improve diagnostic, prognostic, and therapeutic approaches for brain tumors [87]. So this study suggests the potential for further research to develop DNA methylation-targeted drugs in cMGTs.

This study has several limitations that should be considered when interpreting the findings. First, although we applied a genome-wide DNA methylation array, the sample size was relatively small (10 dogs with cMGTs and 8 controls), which limits the statistical power to detect individual differentially methylated CpG sites or genes at a stringent multiple-testing-corrected threshold. In the differential methylation analysis, no single CpG site or gene reached statistical significance after Benjamini–Hochberg correction for multiple comparisons, even though a number of loci showed nominal *P* values below 0.05 and clear differences in effect size (Δ*β*) between groups. Consequently, the gene- and pathway-level results presented here should be regarded as strictly exploratory and hypothesis generating: Candidate hypomethylated and hypermethylated genes were defined on the basis of nominal significance thresholds and effect sizes, and these gene sets were then used for overrepresentation and GO enrichment analyses. The enrichment of 22 GO terms provides a preliminary theoretical framework of plausible biological narratives, but it inherently carries statistical noise and does not establish proven mechanisms of cMGT progression.

Second, this study lacks independent experimental validation, such as bisulfite pyrosequencing, and integrated transcriptomic data (RNA sequencing). Although RNA sequencing was initially considered to directly determine how DNA methylation changes affect mRNA transcription in cMGTs, the inherent instability and degradation of RNA in the stored peripheral blood samples resulted in suboptimal RNA integrity numbers, precluding reliable analysis. This limitation is particularly relevant because, although DNA methylation and gene expression are generally thought to be inversely correlated, there are reports of positive correlations for specific genes [88] and of DNA hypermethylation being associated with up-regulated gene expression in certain cancers, such as human prostate cancer [32]. Consequently, without targeted validation and transcriptomic data, we cannot definitively conclude that the observed DNA methylation changes are free from statistical noise or directly translate into altered mRNA expression.

Third, the study population consisted entirely of client-owned clinical patients from a veterinary hospital rather than purpose-bred experimental animals. In this real-world veterinary setting, extensive clinical staging procedures, biopsies, and the systematic documentation of specific tumor histological subtypes, clinical stage (TNM), and metastasis status were often limited by the owners’ financial constraints and consent. Furthermore, relying on a clinical population precluded the strict control and matching of age and breed between the cMGT and control groups. This demographic variability is an important consideration, as age is a strong driver of genome-wide epigenetic drift, meaning that age-related epigenetic changes may partially confound the observed differential methylation. Despite these practical limitations, the use of peripheral blood DNA methylation profiling to assess tumor-associated systemic epigenetic alterations represents a meaningful, noninvasive step toward investigating prognostic indicators in cMGTs, warranting further large-scale, controlled validation studies.

Fourth, because the animals were client-owned, comprehensive longitudinal follow-up was difficult to achieve. Many patients were lost to follow-up following initial diagnosis or surgical intervention, restricting our ability to gather comprehensive prognostic data and directly correlate specific DNA methylation patterns with long-term clinical outcomes or metastatic progression. Despite these limitations, the hypomethylated genes identified in the cMGT group, compared to the control group, showed patterns that were generally consistent with previous cMGT studies and with reported tumor-associated pathways in human cancers, supporting the biological plausibility of the observed epigenetic changes.

Despite these limitations, the hypomethylated genes identified in the cMGT group, compared to the control group, showed patterns that were generally consistent with previous cMGT studies and with reported tumor-associated pathways in human cancers, supporting the biological plausibility of the observed epigenetic changes. In most previous studies, tissue sampling has been used to investigate protein expression by immunohistochemistry and to evaluate prognosis, whereas the present study explored these associations using a less invasive, blood-based DNA methylation approach. Although further research with larger cohorts, subtype stratification, integrated RNA sequencing, and prospective follow-up is needed to validate prognostic performance and improve accuracy, the use of peripheral blood DNA methylation profiling to assess tumor-associated epigenetic alterations represents a meaningful step toward reducing invasiveness and animal suffering while investigating prognostic indicators in cMGTs.

## Materials and Methods

### Study population and design

Initially, 20 dogs were enrolled in this study, 10 of which were diagnosed with cMGTs, while the other 10 served as the control group. However, 2 control samples were excluded from downstream analyses because they failed to meet the minimum DNA concentration and purity thresholds required for array hybridization during the preanalytical quality assessment. Consequently, the final analysis was performed on a total of 18 dogs, consisting of 10 cMGT dogs and 8 controls (Table 1). Furthermore, due to the clinical nature of the cohort, age and breed could not be strictly matched between the groups, which serves as a potential confounding variable in the epigenetic analyses. The diagnosis of the 10 dogs with cMGTs was confirmed through histological examination. This study was approved by the Animal Ethics Committee and the Institutional Review Board of Chonnam National University (CNU IACUC-YB-2021-166 and CNU IACUC-YB-2024-60).

### DNA methylation analysis

For DNA methylation analysis, 18 dogs with or without cMGTs were selected. Whole blood was collected from each dog’s jugular vein, transferred into an EDTA tube, and stored at −70 °C until extraction of genomic DNA. The genomic DNA was extracted from the buffy coat layer. For DNA methylation profiling, 500 ng of genomic DNA from each sample was bisulfite-converted using the EZ DNA Methylation Kit (Zymo Research, Irvine, CA, USA). The bisulfite-converted DNA (200 ng) was then processed according to the Illumina Infinium HD Methylation Protocol. This involved automated amplification, followed by fragmentation and precipitation of the DNA. The resuspended product was then hybridized onto the Illumina Infinium MethylationEPIC v2.0 BeadChip (Illumina Inc., CA, USA) for 16 h at 37 °C. Considering the high conservation of CpG sites across mammalian species, this human-designed array has been effectively used in canine epigenetic research [89,90]. Based on these studies, we performed DNA methylation profiling of canine samples following the manufacturer’s protocol. To ensure cross-species compatibility and measurement reliability of the human-designed array, raw data were rigorously preprocessed using the SeSAMe v1.20.0 (SEnsible Step-wise Analysis of DNA MEthylation) package. The preprocessing pipeline included dye bias correction and background subtraction using out-of-band probes (noob). Crucially, stringent species-specific quality masking was applied to exclude nonconserved, cross-reactive, or poor-quality probes mapping to the canine reference genome. After this rigorous probe filtering and purification process, 72,768 reliable CpG sites remained and were utilized for all subsequent downstream statistical analyses. The methylation pattern in the blood was confirmed through a heatmap of the 2-way hierarchical clustering analysis performed in the entire group. The heatmap showed the abundance of each methylation (rows) in each sample (column), with yellow indicating high methylation and blue indicating low methylation.

### DMR analysis

Differential methylation analysis was conducted to identify significantly altered CpG sites, using delta mean, odds ratio, fold change, and linear regression methods. A threshold of |delta-mean| ≥ 0.2 and a nominal *P* < 0.05 were set as significance cutoffs.

### Functional annotation

Gene enrichment and functional annotation analysis were carried out using KnowYourCG of SeSAMe [91], gProfiler2 v0.2.3 [92] and clusterProfiler v4.9.0 [93]. Enrichment with Benjamini–Hochberg-adjusted *P* < 0.05 was regarded as significant. Pathway maps with colored delta mean were generated using pathview v1.40.0 [94].

### Statistical analysis and visualization

Principal components analysis was conducted on the transformed gene expression data using the prcomp function from a R core package, stats v3.6.2. The principal components analysis plot, volcano plots, pie chart, bar plots, dot plots, and network plots were generated using ggplot2 v3.4.4. The Venn diagrams and heatmaps were drawn using ggvenn 0.1.10 and ComplexHeatmap v2.16.0 [95]. All the statistical analyses and visualization were performed in R v4.3.2 and R Studio environment.

### Comparison with human dataset

For the comparison with the human dataset, we compared our analysis result with the list of differentially methylated CpG sites identified in the main analysis of normal breast tissue from a previous epigenome-wide study [96]. While cross-species comparisons are traditionally based on tumor-versus-normal datasets, we deliberately selected this dataset because it utilized a nested case-control design comparing histologically normal breast tissue from patients who subsequently developed contralateral breast cancer to matched controls who did not [96].

The rationale for using this specific dataset as a reference is that it captures early, predisposing epigenetic marks indicative of a high-risk state for malignancy, rather than alterations confounded by cancer field effects. This approach directly aligns with our study’s objective of identifying blood-based epigenetic biomarkers for early detection and risk assessment in cMGTs.

Methodologically, rather than reprocessing raw human data, we utilized the validated list of differentially methylated loci from the referenced study, which had been originally normalized and processed using robust pipelines such as the minfi and ENmix packages [96]. For the cross-species comparative analysis, orthologous gene mapping between canines and humans was performed on the basis of official gene symbols to identify shared epigenetic alterations. To visualize set relationships of DMR-associated genes, a Venn diagram and upset plot were drawn using ggvenn v0.1.10 and ComplexHeatmap v2.22.0 [95]. The GO and disease ontology terms representing the functions of the intersected and unique genes were accessed by overrepresentation analysis using clusterProfiler v4.14.4 [93]. The rank correlation between human and dog DMRs were visualized using ggplot2 v3.5.1.

## Ethical Approval

The study protocol was approved by the Institutional Animal Care and Use Committee at Chonnam National University (CNU IACUC-YB-2021-166 and CNU IACUC-YB-2024-60). Owners of all animals gave written, informed consent. All diagnostic procedures were clinically indicated, were to the benefit of the patient, and were performed to the highest standards of veterinary practice.

The authors confirm that all methods were carried out in accordance with relevant guidelines and veterinary regulations, and methods were reported in accordance with the Animal Research: Reporting of In Vivo Experiments (ARRIVE) guidelines as applicable.

## Data Availability

The raw (.idat) and processed DNA methylation data generated in this study have been deposited in the National Center for Biotechnology Information Gene Expression Omnibus database under accession number GSE316492 (https://www.ncbi.nlm.nih.gov/geo/query/acc.cgi?acc=GSE316492). All data are publicly available as of the date of publication. Any other supporting data used in the analysis are available from the corresponding author upon reasonable request.
